# Functional Characterization of Patient-Derived Myotubes Carrying ANO5 and ORAI3 Variants in RYR1-Negative Malignant Hyperthermia Susceptibility

**DOI:** 10.3390/genes17080980

**Published:** 2026-08-20

**Authors:** Hirotsugu Miyoshi, Sachiko Otsuki, Kenshiro Kido, Ayako Sumii, Tsuyoshi Ikeda, Guoqiang Xia, Yuko Noda, Tomomi Ishii, Satoshi Kamiya, Soshi Narasaki, Huei-Ming Yeh, Pei-Lung Chen, Yasuko Ichihara, Keiko Mukaida, Yasuo M. Tsutsumi

**Affiliations:** 1 Department of Anesthesiology and Critical Care, Hiroshima University, Hiroshima 734-8551, Japan; sachi85@hiroshima-u.ac.jp (S.O.); kidoken46@gmail.com (K.K.); batakobatake@gmail.com (A.S.); shiyotsudakei@gmail.com (T.I.); d243940@hiroshima-u.ac.jp (G.X.); nodananoda0724@yahoo.co.jp (Y.N.); tomo1201@hiroshima-u.ac.jp (T.I.); satobo-kamiya@hiroshima-u.ac.jp (S.K.); ijaran7@gmail.com (S.N.); mukaida.keiko1@gmail.com (K.M.); yasuo223@hiroshima-u.ac.jp (Y.M.T.); 2 Department of Anesthesiology, National Taiwan University Hospital, Taipei 100, Taiwan; hmyeh88@gmail.com; 3 Graduate Institute of Medical Genomics and Proteomics, National Taiwan University, Taipei 100, Taiwan; paylong@ntu.edu.tw; 4 Departments of Medical Genetics and Internal Medicine, National Taiwan University Hospital, Taipei 100, Taiwan; 5 Department of Anesthesiology, Kikkoman General Hospital, Chiba 278-0005, Japan; yichihara@nifty.com

**Keywords:** malignant hyperthermia, whole-exome sequencing, calcium homeostasis, skeletal muscle, *ANO5*, *ORAI3*, ryanodine receptor, dantrolene

## Abstract

Background/Objectives: Malignant hyperthermia (MH) is a life-threatening pharmacogenetic disorder of skeletal muscle primarily associated with pathogenic variants in *RYR1*; however, a substantial proportion of MH-susceptible individuals lack identifiable variants in known genes. This study aimed to identify novel genetic contributors in Ca^2+^-induced Ca^2+^ release (CICR)-positive patients without *RYR1* variants and to evaluate their functional relevance. Methods: Among 29 CICR-positive individuals without pathogenic variants identified by gene panel testing, five patients underwent whole-exome sequencing (WES). In one family, heterozygous variants in *ANO5* (p.Arg547Gln) and *ORAI3* (p.Arg287Cys) were identified. To evaluate their functional significance, intracellular Ca^2+^ dynamics were analyzed in primary myotubes derived from Case 165, an affected individual carrying both variants, and compared with those from CICR-negative controls and CICR-positive patients harboring *RYR1* variants. Results: Myotubes derived from Case 165 demonstrated enhanced sensitivity to caffeine and 4-chloro-m-cresol, elevated resting intracellular Ca^2+^ levels, and greater Ca^2+^ reduction under Ca^2+^-free conditions compared with CICR-negative controls, resembling the phenotype observed in the RYR1 variant group. In contrast, dantrolene-induced Ca^2+^ reduction was significantly greater only in the RYR1 variant group. Conclusions: These findings suggest that *ANO5* and *ORAI3* variants may contribute to abnormal Ca^2+^ regulation in MH-susceptible individuals without *RYR1* mutations and highlight the importance of combining genomic analysis with functional validation to identify novel genetic contributors to MH susceptibility.

## 1. Introduction

Malignant hyperthermia (MH) is a rare but life-threatening pharmacogenetic disorder of skeletal muscle, typically triggered by volatile anesthetics and depolarizing neuromuscular blocking agents [1]. Clinically, MH is characterized by a hypermetabolic crisis with rapid increases in end-tidal CO_2_, hyperthermia, acidosis, muscle rigidity, and rhabdomyolysis, which can be fatal without prompt treatment [2]. At the cellular level, MH results from dysregulated intracellular calcium (Ca^2+^) homeostasis in skeletal muscle, leading to sustained Ca^2+^ release from the sarcoplasmic reticulum and uncontrolled contractile and metabolic activation [3]. Because modern anesthesia is widely practiced worldwide, understanding the genetic and molecular basis of MH remains of critical importance for patient safety and precision perioperative medicine.

To date, pathogenic variants in the ryanodine receptor type 1 gene (*RYR1*), which encodes the principal Ca^2+^ release channel of the sarcoplasmic reticulum in skeletal muscle, account for approximately 50–70% of MH-susceptible (MHS) cases [4,5]. In addition, variants in *CACNA1S*, encoding the α1S subunit of the dihydropyridine receptor (DHPR), have been identified in approximately 1–2% of cases [6]. These two proteins are key components of excitation–contraction coupling, physically and functionally interacting to mediate depolarization-induced Ca^2+^ release [7]. However, in approximately 30–40% of clinically diagnosed MHS individuals, no pathogenic variants are identified in *RYR1* or *CACNA1S* [8]. This substantial proportion of genetically unresolved cases suggests that additional genes may contribute to MH susceptibility. Identifying additional genetic contributors is therefore essential to improve molecular diagnosis and to better understand the mechanisms underlying MH susceptibility.

The regulation of intracellular Ca^2+^ in skeletal muscle is a complex, highly coordinated process involving not only RYR1 and DHPR, but also accessory proteins, luminal Ca^2+^-binding proteins, membrane channels, and Ca^2+^ reuptake systems [9]. Variants in genes encoding these components could theoretically alter Ca^2+^ release, buffering, or reuptake, thereby predisposing individuals to aberrant Ca^2+^ signaling under anesthetic exposure. Indeed, emerging genetic studies and next-generation sequencing approaches have proposed several candidate genes beyond *RYR1* and *CACNA1S*; however, their pathogenic roles remain controversial and often lack functional validation [10,11]. One major challenge in the field is distinguishing rare benign variants from truly causative mutations, particularly in the absence of robust in vitro or in vivo functional data [12]. Thus, comprehensive genetic analysis combined with functional assays that directly assess Ca^2+^ dynamics is essential to clarify the pathogenic relevance of novel variants.

In this study, we sought to identify novel candidate variants in MH-susceptible individuals without known MH-related mutations using advanced genetic analysis. To determine their potential involvement in MH pathogenesis, we performed functional evaluation by analyzing intracellular Ca^2+^ dynamics in cultured skeletal muscle cells (myotubes). We demonstrate that selected variants are associated with altered Ca^2+^ handling properties consistent with an MH-like phenotype, supporting their possible contribution to disease susceptibility. These findings expand the spectrum of genes potentially involved in MH and provide further insight into the molecular mechanisms underlying dysregulated Ca^2+^ homeostasis in skeletal muscle.

## 2. Materials and Methods

### 2.1. Ethics and Study Participants

The study was approved by the institutional ethics committee (Approval No. E2015-9151-26), and all raw sequencing data obtained from the gene panel testing were submitted to the DNA Data Bank of Japan (DDBJ) Sequence Read Archive (DRA) under accession number BioProject: PRJDB35521. Written informed consent was obtained from all participants who underwent the Ca^2+^-induced Ca^2+^ release (CICR) test, all participants who underwent whole-exome sequencing (WES), and participants who underwent gene panel testing. For a subset of previously collected archived samples used for gene panel testing, when re-consent could not be obtained because participants were unreachable despite reasonable efforts or had died, the study was conducted under an institutional ethics committee-approved opt-out consent procedure in accordance with institutional guidelines.

MH-susceptible (MHS) individuals were selected from patients registered in the Hiroshima University database who had been referred for evaluation of malignant hyperthermia susceptibility; among these, those with a positive CICR test using surgically obtained skeletal muscle specimens and in whom no pathogenic or likely pathogenic variants in known MH-related genes (including *RYR1* and *CACNA1S*) were identified by conventional gene panel testing subsequently underwent WES.

**Figure 1:**
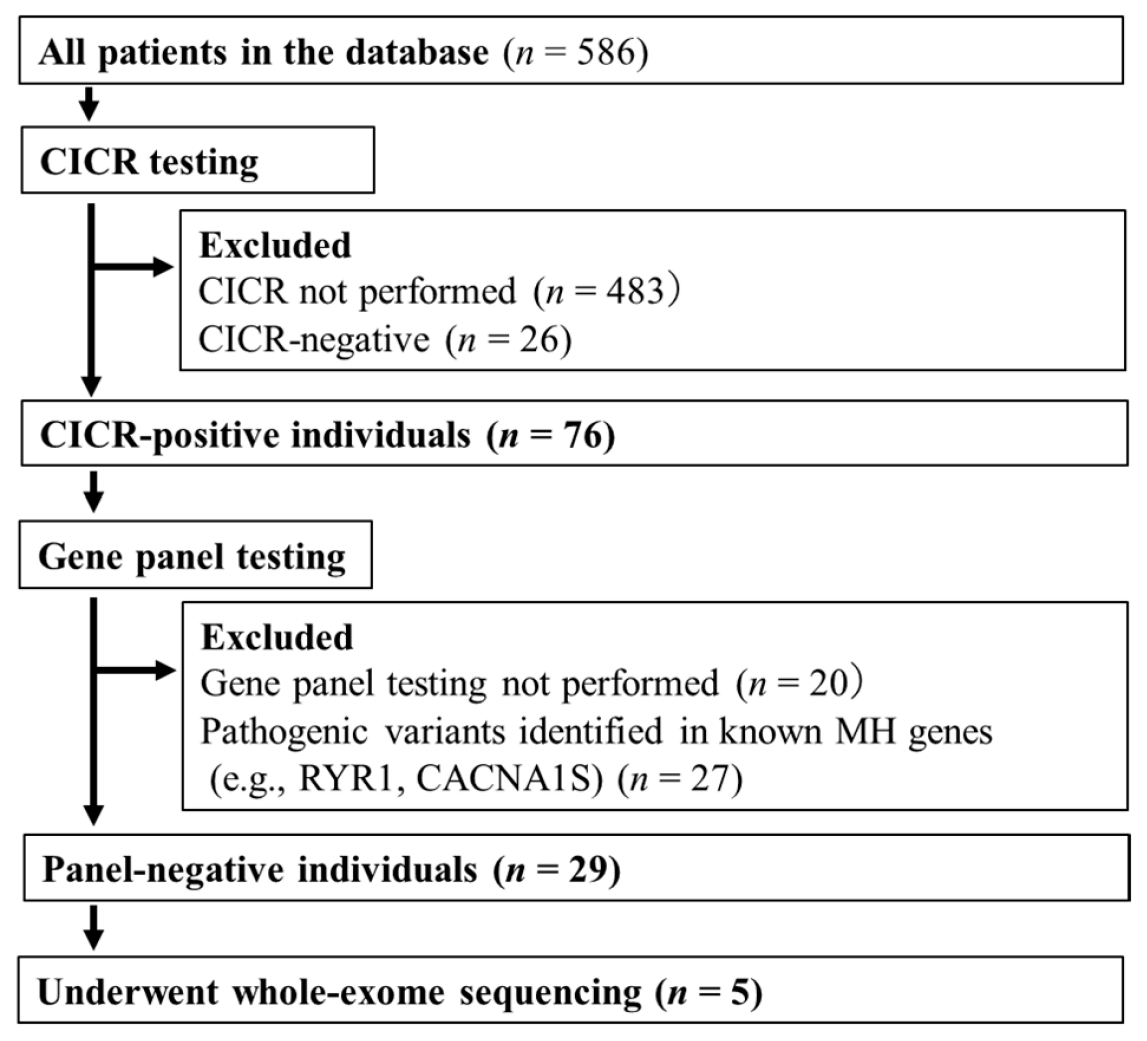
Patient selection criteria.

Patients registered in the Hiroshima University database who were referred for evaluation of malignant hyperthermia (MH) susceptibility were screened. Individuals with a positive Ca^2+^-induced Ca^2+^ release (CICR) test and no pathogenic or likely pathogenic variants identified by conventional MH-related gene panel testing were considered eligible for further genetic investigation. Among 29 eligible MH-susceptible (MHS) individuals, five unrelated patients with well-defined phenotypes and high-quality biological samples were selected for WES and subsequent functional analyses. Patient selection criteria are shown in Figure 1.

### 2.2. CICR Test

Skeletal muscle samples were obtained by biopsy from the quadriceps or biceps brachii muscles. To prepare skinned fibers, the muscle cell membranes were chemically permeabilized using saponin, and isometric tension was recorded for each specimen with a force transducer. The CICR assay was conducted in accordance with the method described by Endo et al. [13,14]. Ca^2+^ release rates were assessed in solutions containing five different Ca^2+^ concentrations (0, 0.3, 1.0, 3.0, and 10.0 μM). The degree of CICR acceleration was evaluated as previously reported [15,16].

Reference values were established from 12 individuals with negative IVCT and CHCT results (controls). An accelerated CICR rate, defined as a value exceeding the control mean by more than two standard deviations (SD), was considered indicative of MH susceptibility. Because CICR activity is enhanced in MH-susceptible individuals, patients showing an accelerated rate were classified as “CICR-positive,” whereas those with rates comparable to controls were classified as “CICR-negative.”

### 2.3. Whole-Exome Sequencing and Variant Filtering

#### 2.3.1. DNA Extraction and Sequencing

Peripheral venous blood (5–10 mL) was collected in tubes containing EDTA as an anticoagulant. Samples were kept at 4 °C prior to DNA extraction, and isolated genomic DNA was stored at −20 °C until further analysis. When available, residual skeletal muscle tissue obtained during CICR testing was also used as a source of genomic DNA. DNA was extracted from either whole blood or muscle specimens using the QIAamp DNA Blood Mini Kit (Qiagen GmbH, Cologne, Germany) according to the manufacturer’s instructions. DNA quantity and purity were evaluated with a NanoDrop spectrophotometer (Thermo Fisher Scientific, Waltham, MA, USA). Array-CGH was not performed prior to WES.

#### 2.3.2. Variant Filtering Strategy

Variant filtering was performed using a stepwise approach. First, variants with a minor allele frequency (MAF) ≥ 0.001 in gnomAD v4.0 were excluded. We then focused on protein-altering variants, including nonsense, frameshift, and missense variants. Missense variants were retained only if they fulfilled at least two of the following in silico pathogenicity prediction criteria: a CADD score > 20, a SIFT score ≤ 0.05, and a PolyPhen-2 score > 0.85 [17].

**Figure 2:**
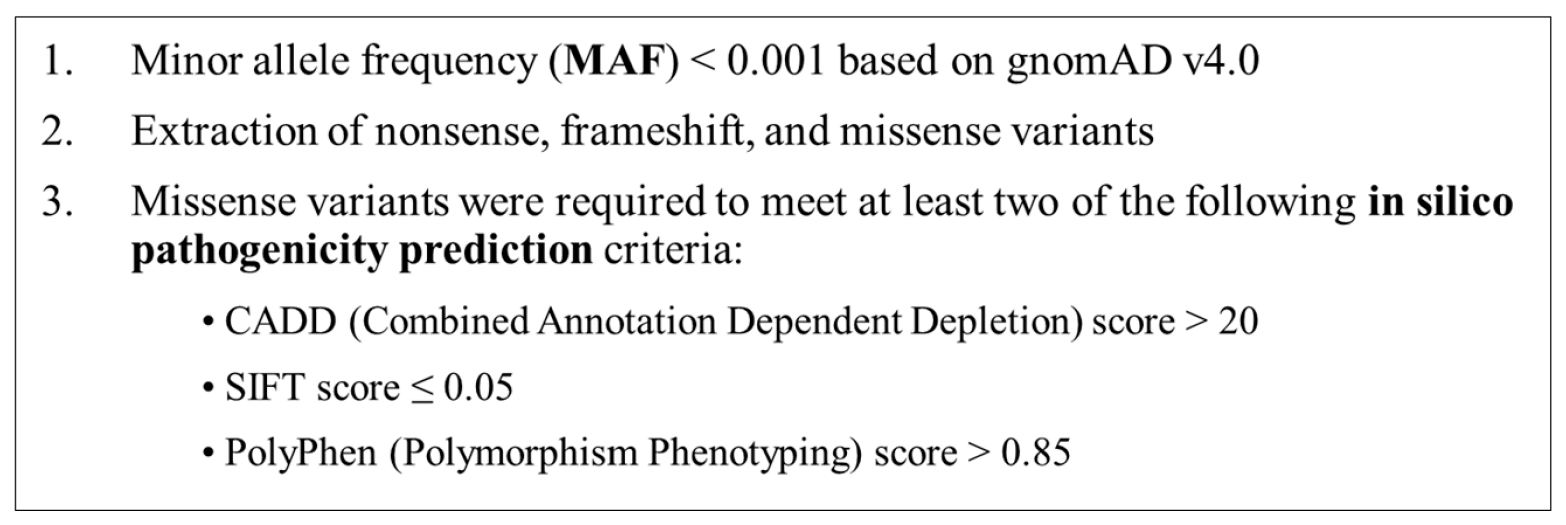
Whole-exome sequencing variant filtering.

Subsequently, variants were prioritized according to gene function, with particular emphasis on genes involved in Ca^2+^ handling, excitation–contraction coupling, sarcoplasmic reticulum function, and store-operated Ca^2+^ entry (SOCE). An overview of the variant filtering and prioritization strategy is summarized in Figure 2.

### 2.4. Myotube Preparation

Following the completion of the CICR rate assay, residual skeletal muscle fibers not subjected to testing were used for primary cell culture. The tissue was maintained in DMEM/F12 medium (Gibco, Waltham, MA, USA) supplemented with 10% heat-inactivated fetal bovine serum (FBS; Gibco, USA), 1% ampicillin sodium salt, 1% kanamycin sulfate (Sigma-Aldrich, St. Louis, MO, USA), and 2.5 μg/mL amphotericin B (Invitrogen, Waltham, MA, USA). Samples were cultured in 25-cm^2^ flasks (Corning, New York, NY, USA) at 37 °C in a humidified incubator with 5% CO_2_, and the medium was replaced every three days.

After 2–3 weeks, proliferating cells were detached using trypsin and transferred onto 35 mm glass-bottom dishes. Cells were further maintained in DMEM/F12 containing 10% FBS for 7–10 days. Myogenic differentiation was subsequently induced by reducing the serum concentration to 2% FBS for 5–7 days. Myotube formation was verified morphologically by identifying elongated, multinucleated cells as previously described [14].

### 2.5. Intracellular Ca^2+^ Measurements

Myotubes were first equilibrated in Hank’s balanced salt solution (HBSS; pH 7.4, 24–26 °C) composed of 130 mM NaCl, 5.4 mM KCl, 20 mM HEPES, 2.5 mM CaCl_2_, 1 mM MgCl_2_, and 5.5 mM glucose. Cells were then incubated with 5.0 μM Fura-2/AM (Dojindo Molecular Technologies, Tokyo, Japan) dissolved in HBSS for 60 min at room temperature, followed by washing with fresh HBSS. Fluorescence measurements were initiated after an additional 30 min stabilization period in HBSS.

Intracellular Ca^2+^ dynamics were monitored using dual-wavelength excitation at 340 and 380 nm, and emission was recorded at 510 nm every 5 s with a fluorescence microscope (Nikon, Tokyo, Japan). The imaging system consisted of a 10× ocular lens and a 10× objective lens, and images were captured using a cooled high-speed digital camera (ORCA-AG; Hamamatsu Photonics, Hamamatsu, Japan). The ratio of fluorescence intensity at 340/380 nm was calculated using a Ca^2+^ imaging analysis system (Aquacosmos 2.5; Hamamatsu Photonics).

Agonist solutions were applied by perfusion, with the test solution introduced from one side of the dish and removed from the opposite side at a flow rate of 1.2 mL/min at 36–37 °C for 2 min. After each application, cells were washed for 2 min before exposure to the next concentration. Dose-dependent Ca^2+^ responses were assessed using increasing concentrations of caffeine (0.25–20 mM) and 4-chloro-m-cresol (3–1000 μM). Cells exhibiting an increase in intracellular Ca^2+^ upon exposure to 10 mM caffeine were considered to express RYR1.

Intracellular Ca^2+^ concentration ([Ca^2+^]_i_) was determined using a commercially available Fura-2 calcium imaging calibration kit (F-6774; Thermo Fisher Scientific, Waltham, MA, USA). The minimum (Rmin) and maximum (Rmax) fluorescence ratios were determined according to the manufacturer’s protocol, and the measured 340/380 fluorescence ratios were converted to intracellular Ca^2+^ concentrations ([Ca^2+^]_i_). For concentration–response analysis, the increase in the 340/380 fluorescence ratio from baseline following agonist stimulation was calculated. Responses at each concentration were normalized to the maximal response induced by caffeine or 4-chloro-m-cresol (4-CmC) in the same cell and expressed as a percentage. Concentration–response curves were generated using GraphPad Prism (GraphPad Software, San Diego, CA, USA), and EC_50_ values were calculated by nonlinear regression.

### 2.6. Ca^2+^ Imaging Protocols

Intracellular Ca^2+^ handling properties were compared among three groups: (1) a negative control group consisting of seven patients with negative CICR results; (2) a positive control group comprising seven patients with positive CICR results harboring pathogenic or likely pathogenic RYR1 variants; and (3) one CICR-positive patient without an identified RYR1 variant who subsequently underwent whole-exome sequencing (WES). The clinical characteristics of these subjects are summarized in Table 1.

The following parameters were evaluated: (A) concentration–response relationships for caffeine and 4-chloro-m-cresol (4-CmC); (B) resting intracellular Ca^2+^ levels; (C) the reduction in intracellular Ca^2+^ during perfusion with a Ca^2+^-free solution; (D) the reduction in intracellular Ca^2+^ following exposure to 50 µM dantrolene; and (E) intracellular Ca^2+^ responses to caffeine during perfusion with a Ca^2+^-free solution. Resting intracellular Ca^2+^ levels were measured after stabilization of the baseline under normal extracellular Ca^2+^ conditions. To assess the contribution of extracellular Ca^2+^ influx, the perfusion solution was then replaced with a Ca^2+^-free solution, and the maximal reduction in intracellular Ca^2+^ from the baseline was quantified. Similarly, after exposure to 50 µM dantrolene, the maximal reduction in intracellular Ca^2+^ from the baseline was measured. For both the Ca^2+^-free solution and dantrolene experiments, the peak decrease in intracellular Ca^2+^ relative to the baseline immediately before each intervention was used for statistical analysis.

### 2.7. Statistical Analysis

The responsiveness of the myotubes to agonists was quantified as the half-maximal effective concentration (EC_50_). Changes in intracellular Ca^2+^ were calculated as the difference between the peak response and the preceding baseline value. For concentration–response analyses, fluorescence ratios were normalized to the maximal response of each cell. EC_50_ values were derived from the fitted concentration–response curves.

Comparisons among the three groups were performed using the Kruskal–Wallis test, followed by Dunn’s multiple comparisons test. Statistical analyses were conducted using GraphPad Prism version 9.0 (GraphPad Software, San Diego, CA, USA). A *p* value < 0.05 was considered statistically significant.

## 3. Results

### 3.1. Patients

**Figure 3:**
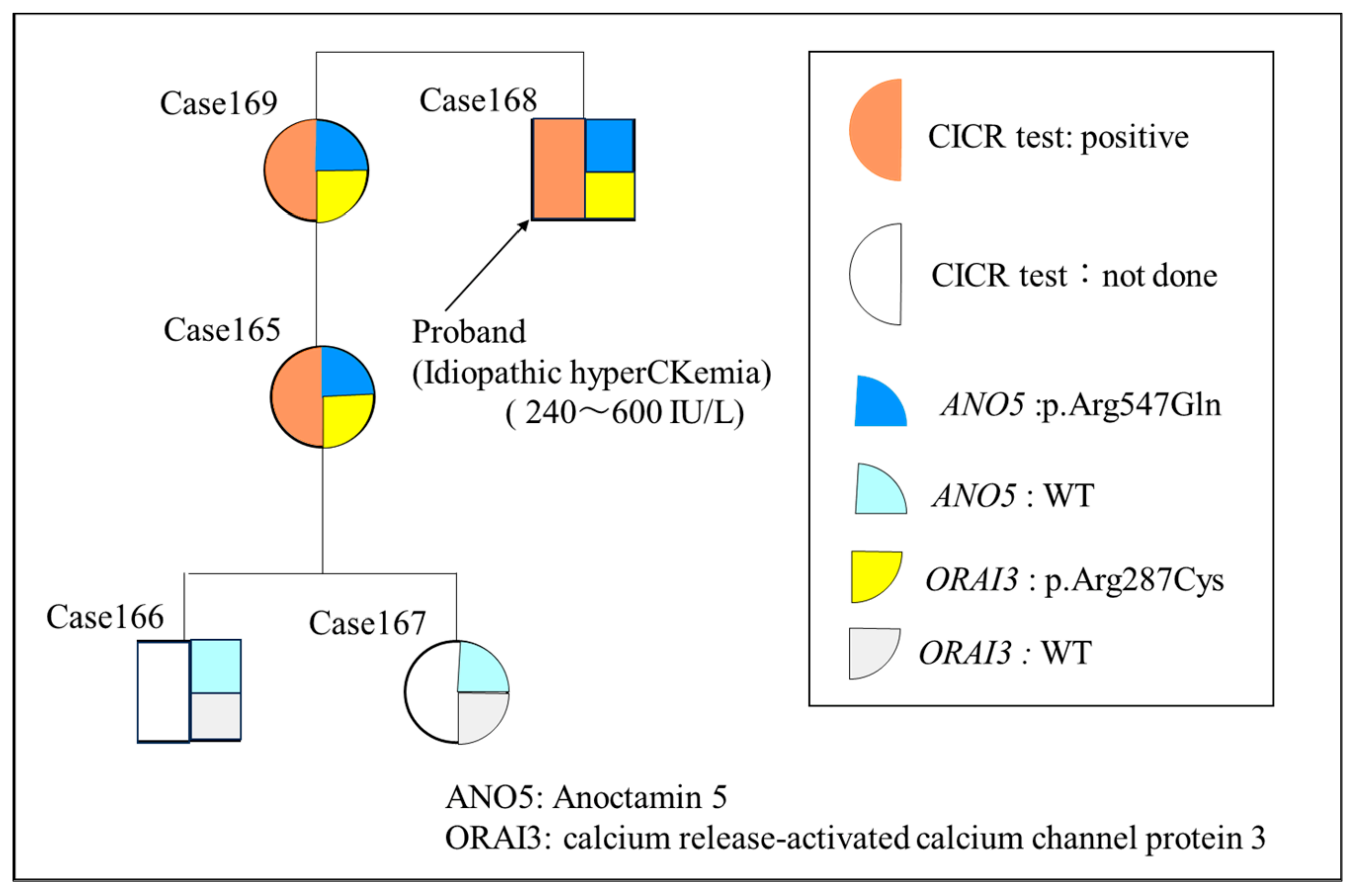
Pedigree of the family with the identified variant.

Whole-exome sequencing was performed in five CICR-positive patients without identified *RYR1* variants. In one family comprising two affected individuals, heterozygous variants in *ANO5* (p.Arg547Gln) and *ORAI3* (p.Arg287Cys) were identified. The familial segregation of these variants is illustrated in Figure 3.

### 3.2. Intracellular Ca^2+^ Handling in Primary Myotubes

Intracellular Ca^2+^ dynamics were compared among three groups: a CICR-negative group (negative control), a CICR-positive group harboring pathogenic RYR1 variants (positive control), and a CICR-positive group without identified RYR1 variants who underwent whole-exome sequencing (WES group). The WES group originally included two individuals, Case 165 and Case 169; however, only the data from Case 165 were available for analysis. Therefore, the WES group ultimately consisted of Case 165 alone.

#### 3.2.1. Concentration–Response Curves (EC_50_)

**Figure 4:**
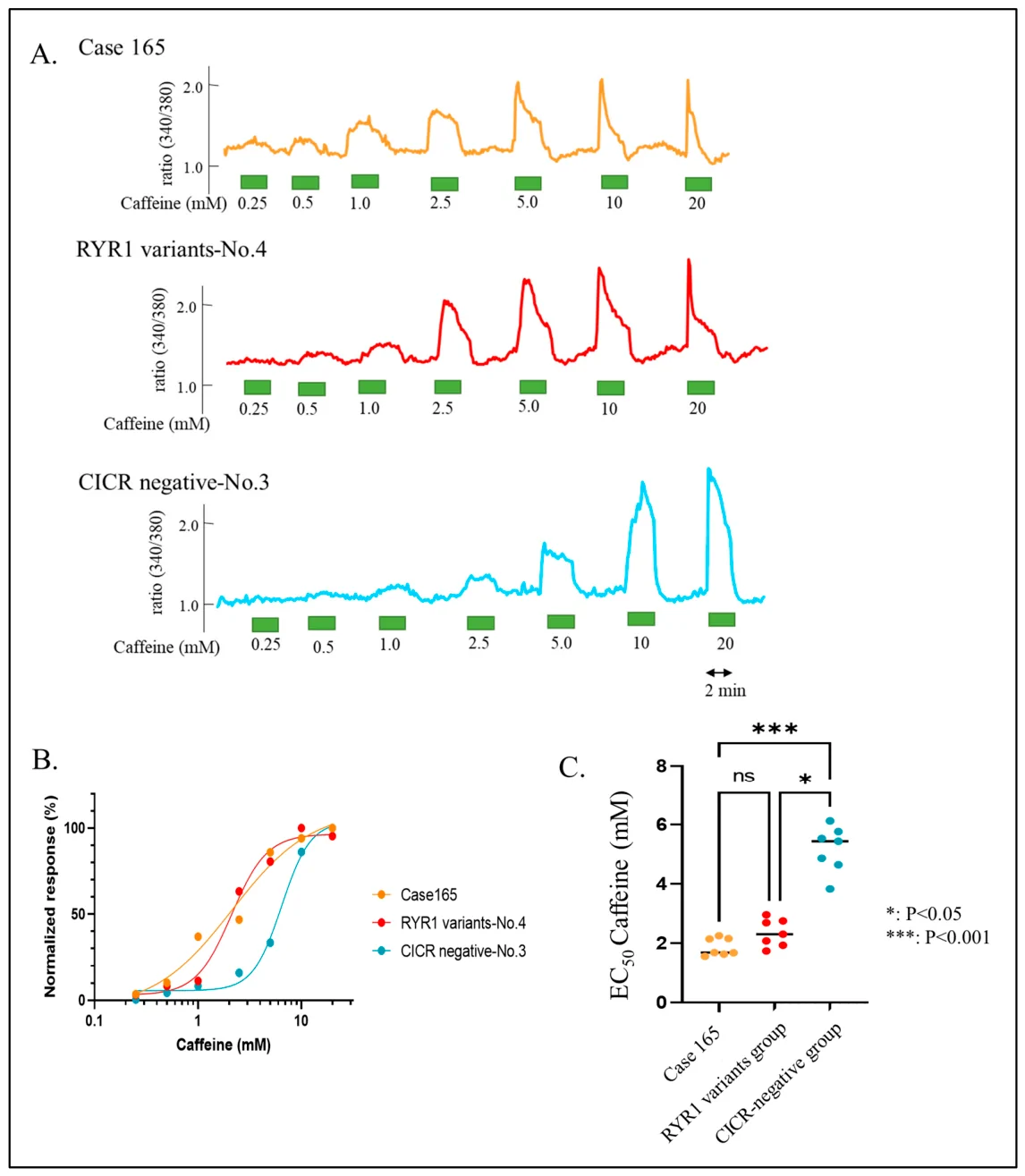
Representative Ca^2+^ responses to caffeine, dose–response curves, and comparison of EC_50_ values among the three groups. (**A**) Representative intracellular Ca^2+^ responses to increasing concentrations of caffeine in cultured myotubes from Case 165, the RYR1 variant group, and the CICR-negative group. Green bars indicate the timing of caffeine application. (**B**) Caffeine concentration–response curves for Case 165, the RYR1 variant group, and the CICR-negative group. (**C**) Comparison of EC_50_ values for caffeine among Case 165, the RYR1 variant group, and the CICR-negative group. Each dot represents one cultured myotube, and horizontal bars indicate the median. Statistical significance was assessed using the Kruskal–Wallis test followed by Dunn’s multiple comparisons test. *: *p* < 0.05, ***: *p* < 0.001, ns: not significant.

**Figure 5:**
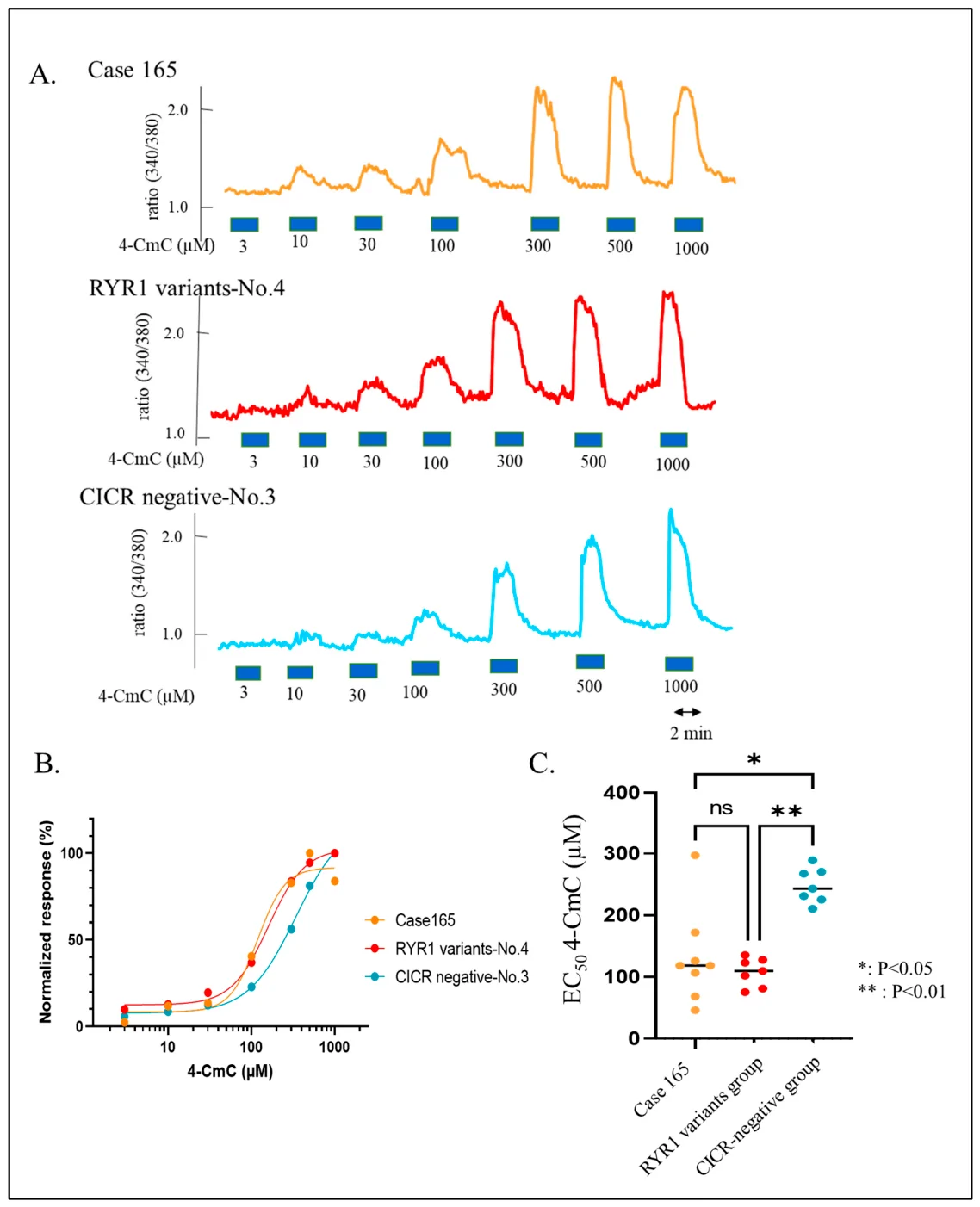
Representative Ca^2+^ responses to 4-CmC, dose–response curves, and comparison of EC_50_ values among the three groups. (**A**) Representative intracellular Ca^2+^ responses to increasing concentrations of 4-CmC in cultured myotubes from Case 165, the RYR1 variant group, and the CICR-negative group. Blue bars indicate the timing of 4-CmC application. (**B**) 4-CmC concentration–response curves for Case 165, the RYR1 variant group, and the CICR-negative group. (**C**) Comparison of EC_50_ values for 4-CmC among Case 165, the RYR1 variant group, and the CICR-negative group. Each dot represents one cultured myotube. Statistical significance was assessed using the Kruskal–Wallis test followed by Dunn’s multiple comparisons test. *: *p* < 0.05, **: *p* < 0.01, ns: not significant.

The EC_50_ values for caffeine and 4-chloro-m-cresol (4-CmC) differed significantly among the three groups. Case 165 demonstrated enhanced sensitivity to both agonists, with EC_50_ values comparable to those observed in the RYR1 variant group and significantly lower than those of the CICR-negative group (Kruskal–Wallis test followed by Dunn’s multiple comparisons test). Representative Ca^2+^ responses to caffeine, the corresponding dose–response curves, and comparisons of EC_50_ values among the three groups are shown in Figure 4. Representative Ca^2+^ responses to 4-CmC, the corresponding dose–response curves, and comparisons of EC_50_ values among the three groups are shown in Figure 5.

#### 3.2.2. Resting Intracellular Ca^2+^ Levels

**Figure 6:**
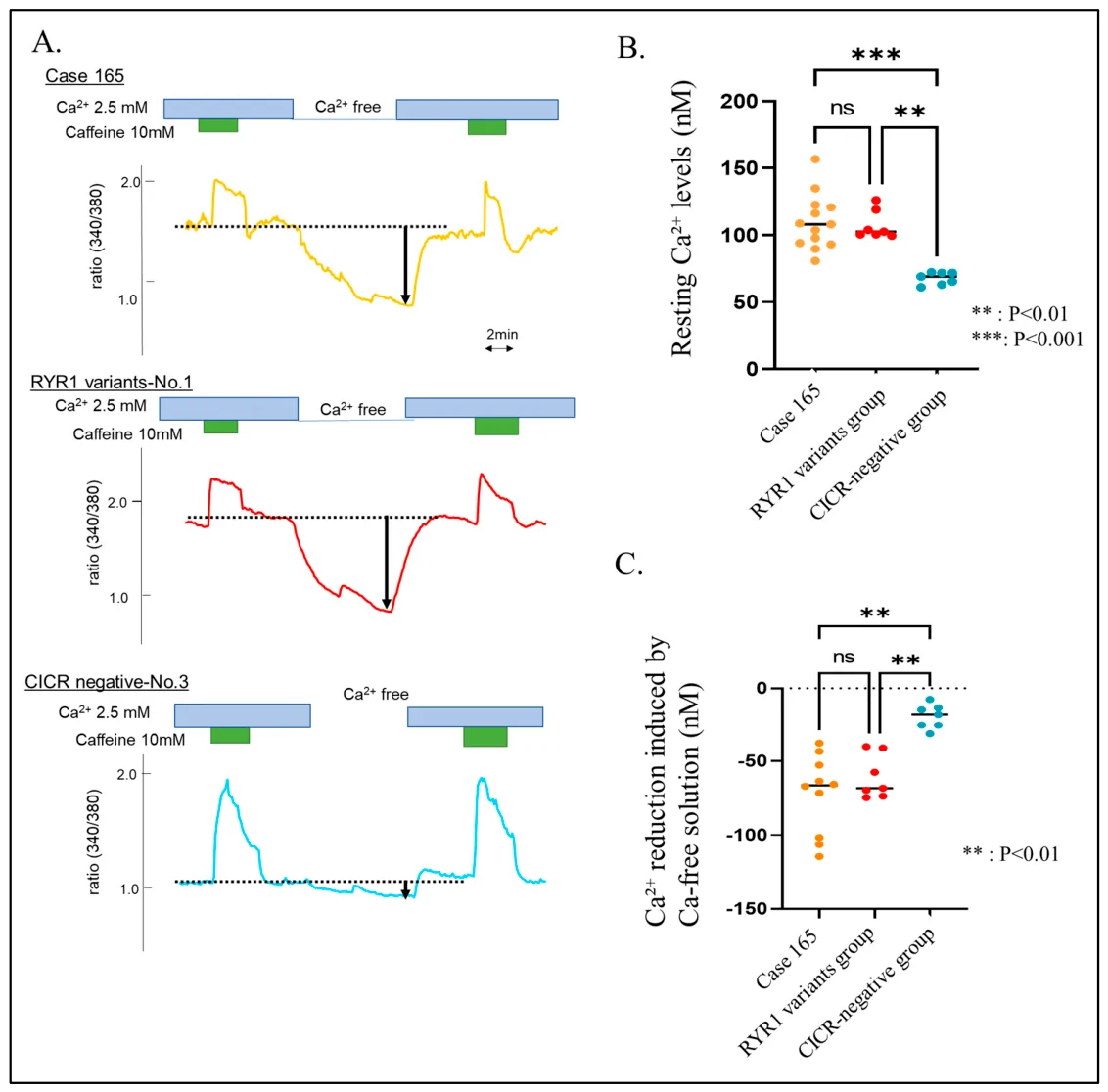
Representative intracellular Ca^2+^ responses to extracellular Ca^2+^ removal and comparison of intracellular Ca^2+^ levels among the three groups. (**A**) Representative intracellular Ca^2+^ traces before and during perfusion with a Ca^2+^-free solution in cultured myotubes. Light blue bars indicate perfusion with Ca^2+^-containing solution, the interval between the bars indicates perfusion with Ca^2+^-free solution, and green bars indicate caffeine exposure. The dashed horizontal line indicates the reference level for resting intracellular Ca^2+^, and the black arrows indicate the magnitude of the change in intracellular Ca^2+^ induced by perfusion with the Ca^2+^-free solution. (**B**) Comparison of resting intracellular Ca^2+^ levels among Case 165, the RYR1 variant group, and the CICR-negative group. (**C**) Comparison of intracellular Ca^2+^ levels during perfusion with Ca^2+^-free solution among Case 165, the RYR1 variant group, and the CICR-negative group. Each dot represents one cultured myotube. Statistical significance was assessed using the Kruskal–Wallis test followed by Dunn’s multiple comparisons test. **: *p* < 0.01, ***: *p* < 0.001, ns: not significant.

Basal intracellular Ca^2+^ concentrations were significantly elevated in both Case 165 and the *RYR1* variant group compared with the CICR-negative group, whereas no significant difference was observed between Case 165 and *RYR1* variant groups. The results are shown in Figure 6B.

#### 3.2.3. Ca^2+^ Reduction Induced by Ca^2+^-Free Solution

Perfusion with Ca^2+^-free solution resulted in a significantly greater decline in intracellular Ca^2+^ in Case 165 and the RYR1 variant group compared with the CICR-negative group. The magnitude of Ca^2+^ reduction did not differ significantly between Case 165 and RYR1 variant groups. The results are shown in Figure 6C. These findings indicate that extracellular Ca^2+^ contributes to maintenance of the elevated resting intracellular Ca^2+^ level in Case 165 and RYR1 variant groups.

#### 3.2.4. Ca^2+^ Reduction Induced by Dantrolene

**Figure 7:**
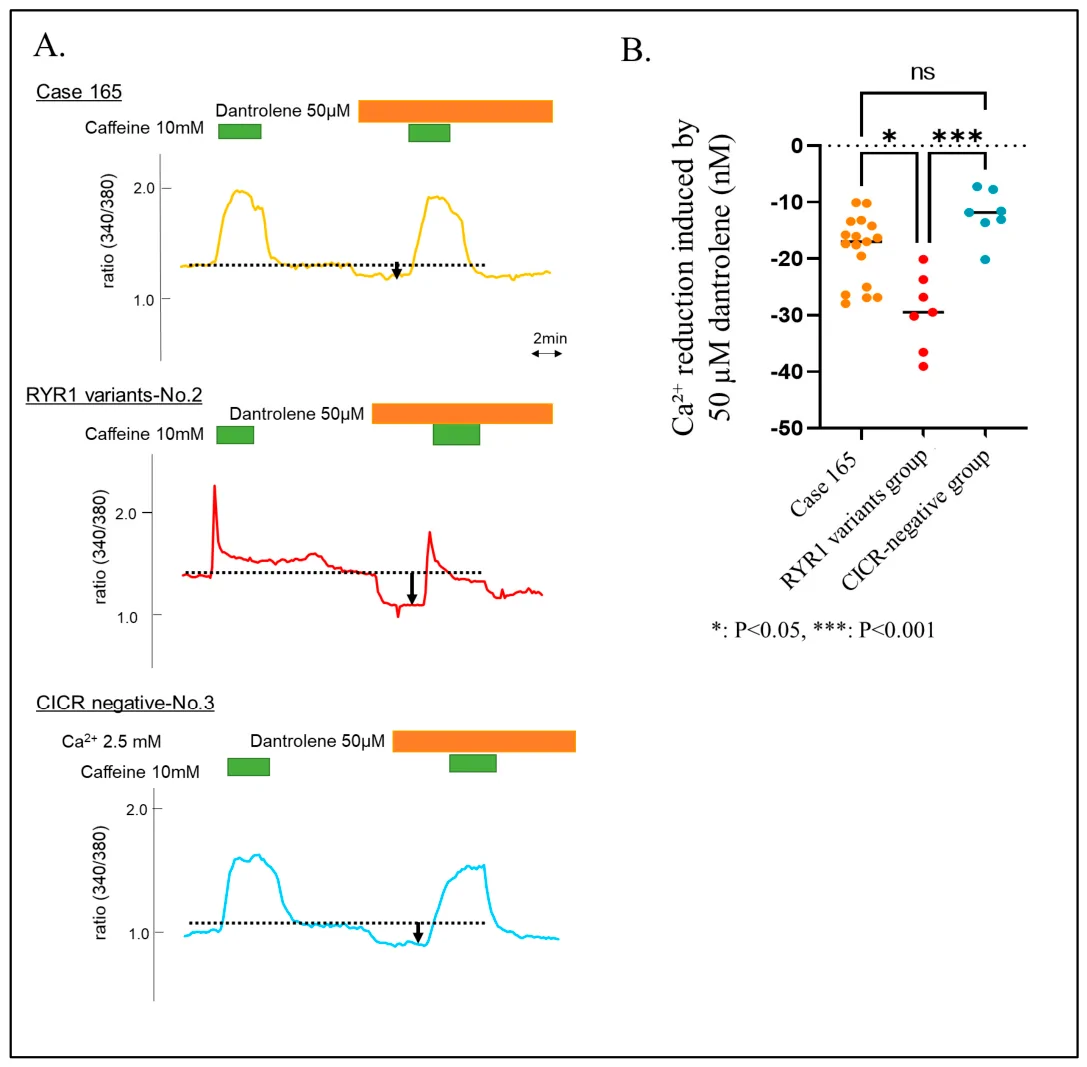
Ca^2+^ Reduction Induced by Dantrolene. (**A**) Representative intracellular Ca^2+^ traces showing the responses to caffeine and subsequent treatment with 50 μM dantrolene in cultured myotubes from Case 165, the RYR1 variant group, and the CICR-negative group. Green bars indicate caffeine exposure, and orange bars indicate exposure to 50 μM dantrolene. The dashed horizontal line indicates the baseline intracellular Ca^2+^ level, and the black arrows indicate the reduction in intracellular Ca^2+^ from the baseline following dantrolene treatment. (**B**) Comparison of the reduction in intracellular Ca^2+^ induced by 50 μM dantrolene among Case 165, the RYR1 variant group, and the CICR-negative group. Each dot represents one cultured myotube. Statistical significance was assessed using the Kruskal–Wallis test followed by Dunn’s multiple comparisons test. *: *p* < 0.05, ***: *p* < 0.001, ns: not significant.

Exposure to 50 µM dantrolene resulted in a significantly greater reduction in intracellular Ca^2+^ in the RYR1 variant group compared with both Case 165 and the CICR-negative group. No significant difference was observed between Case 165 and the CICR-negative group. This result suggests that RyR1-mediated Ca^2+^ release contributes less prominently to the abnormal Ca^2+^ homeostasis in Case 165 than in the RYR1 variant group. The results are shown in Figure 7.

#### 3.2.5. Intracellular Ca^2+^ Responses to Caffeine During Perfusion with a Ca^2+^-Free Solution

**Figure 8:**
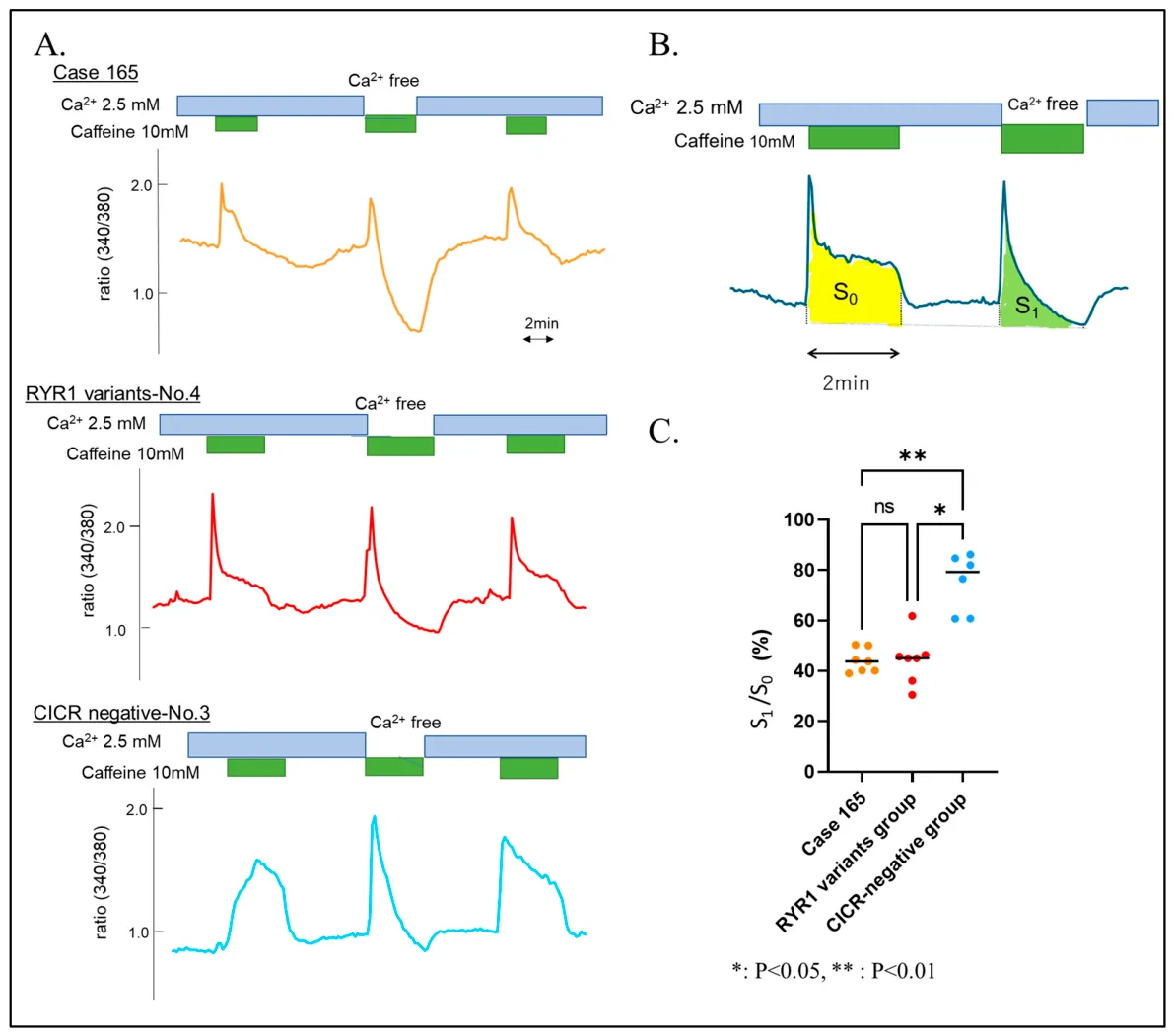
Intracellular Ca^2+^ responses to caffeine during perfusion with a Ca^2+^-free solution. (**A**) Representative intracellular Ca^2+^ traces from the RYR1 variant group (orange), Case 165 (red), and the CICR-negative group (cyan). Light blue bars indicate perfusion with Ca^2+^-containing solution, the interval between the bars indicates perfusion with Ca^2+^-free solution, and green bars indicate caffeine exposure. (**B**) Schematic of the experimental protocol. The areas under the first and second caffeine-induced Ca^2+^ transients were defined as S_0_ and S_1_, respectively, and the S_1_/S_0_ ratio was calculated. (**C**) Comparison of the S_1_/S_0_ ratio among the RYR1 variant group, Case 165, and the CICR-negative group. Each dot represents an individual experiment, and horizontal bars indicate the mean. Statistical significance was assessed using the Kruskal–Wallis test followed by Dunn’s multiple comparisons test. *: *p* < 0.05, **: *p* < 0.01, ns: not significant.

The areas under the intracellular Ca^2+^ transients induced by caffeine in the presence of extracellular Ca^2+^ (S_0_) and during subsequent perfusion with Ca^2+^-free solution (S_1_) were measured, and the S_1_/S_0_ ratio was calculated. The S_1_/S_0_ ratio was significantly lower in both the RYR1 variant group and Case 165 than in the CICR-negative group. No significant difference was observed between the RYR1 variant group and Case 165. These findings indicate that repeated caffeine-induced Ca^2+^ release in Case 165 is more dependent on extracellular Ca^2+^ than in CICR-negative controls. The results are shown in Figure 8.

#### 3.2.6. Comparison of Intracellular Ca^2+^ Handling Parameters Among the Three Study Groups

A summary of intracellular Ca^2+^ handling parameters across the three groups is presented in Table 2. Overall, myotubes derived from Case 165 exhibited Ca^2+^ regulatory properties largely comparable to those observed in the RYR1 variant group and distinct from those of the CICR-negative group.

Specifically, the median EC_50_ values for both caffeine and 4-CmC were significantly lower in Case 165 and RYR1 variant groups than in the CICR-negative group. Resting intracellular Ca^2+^ concentrations were elevated in Case 165 and RYR1 variant groups compared with the CICR-negative group. Similarly, the magnitude of Ca^2+^ reduction induced by Ca^2+^-free solution was significantly greater in Case 165 and RYR1 variant groups than in the CICR-negative group, with no significant difference between Case 165 and RYR1 variant groups.

In contrast, exposure to 50 µM dantrolene resulted in a significantly greater reduction in intracellular Ca^2+^ in the RYR1 variant group compared with both Case 165 and CICR-negative groups, whereas no significant difference was observed between Case 165 and CICR-negative groups.

Taken together, these findings indicate that Ca^2+^ dysregulation in Case 165 resembles the RYR1-associated phenotype in most parameters, although the response to dantrolene differed from that of the RYR1 variant group.

## 4. Discussion

Malignant hyperthermia (MH) is a life-threatening pharmacogenetic disorder characterized by dysregulated intracellular Ca^2+^ homeostasis in skeletal muscle and is most commonly associated with pathogenic variants in *RYR1* [1,8]. However, approximately 30–40% of individuals with MH susceptibility lack identifiable variants in known MH-related genes, suggesting additional genetic contributors [2].

In the present study, whole-exome sequencing in CICR-positive individuals without pathogenic variants detected by gene panel testing identified heterozygous variants in *ANO5* and *ORAI3* in one family. Functional evaluation using patient-derived myotubes demonstrated abnormal intracellular Ca^2+^ regulation comparable to that observed in RYR1-associated MH.

Case 165 exhibited enhanced sensitivity to caffeine and 4-CmC, elevated resting intracellular Ca^2+^ levels, and greater Ca^2+^ reduction under Ca^2+^-free conditions compared with the CICR-negative group. These findings are consistent with a hyperactive Ca^2+^ signaling phenotype, a hallmark of MH pathophysiology [1,18]. Notably, these abnormalities closely resembled those observed in the RYR1 variant group, suggesting that the identified variants may contribute to MH susceptibility through dysregulation of intracellular Ca^2+^ handling.

*ANO5* encodes anoctamin-5, a protein localized predominantly to intracellular membranes, including the sarcoplasmic reticulum, and has been implicated in the regulation of skeletal muscle membrane repair and Ca^2+^ homeostasis [19]. Mutations in *ANO5* are associated with muscular dystrophy phenotypes, in which impaired Ca^2+^ handling has been proposed as a pathogenic mechanism [19,20]. The *ANO5* variant identified in this study (p.Arg547Gln) has previously been reported in association with limb-girdle muscular dystrophy (LGMD) and related myopathic phenotypes [21]. Therefore, this variant cannot be considered novel. However, to the best of our knowledge, its association with malignant hyperthermia (MH) susceptibility has not been reported. The identification of this previously described ANO5 variant in a RYR1-negative, CICR-positive family raises the possibility that ANO5 may contribute to abnormal Ca^2+^ regulation in a subset of MH-susceptible individuals.

*ORAI3* encodes a plasma membrane Ca^2+^ channel that functions as a component of store-operated Ca^2+^ entry (SOCE), a pathway critical for replenishing intracellular Ca^2+^ stores following sarcoplasmic reticulum depletion [22,23]. SOCE has been increasingly recognized as an important modulator of skeletal muscle Ca^2+^ signaling, and its dysregulation may contribute to abnormal Ca^2+^ dynamics [23,24]. One possible explanation is that the *ORAI3* p.Arg287Cys variant alters the regulation of SOCE, a mechanism that replenishes intracellular Ca^2+^ stores by promoting extracellular Ca^2+^ influx after sarcoplasmic reticulum (SR) Ca^2+^ depletion. If so, increased dependence on this pathway could account for the greater decrease in resting intracellular Ca^2+^ observed after extracellular Ca^2+^ removal and the lower S_1_/S_0_ ratio in Case 165. Although SOCE activity was not directly measured in the present study, these findings are consistent with the possibility that altered ORAI3 function contributes to abnormal extracellular Ca^2+^ entry.

As shown in Figure 6A, resting intracellular Ca^2+^ levels were elevated in myotubes derived from Case 165 as well as in those from patients harboring pathogenic *RYR1* variants compared with CICR-negative controls. Furthermore, the removal of extracellular Ca^2+^ reduced resting intracellular Ca^2+^ levels in both Case 165 and the RYR1 variant group (Figure 6C), suggesting that sustained Ca^2+^ influx from the extracellular space contributes to the elevated resting intracellular Ca^2+^ concentration. Consistent with these observations, the S_1_/S_0_ ratio was significantly lower in both Case 165 and the RYR1 variant group than in the CICR-negative group (Figure 8), further supporting the notion that the elevated resting intracellular Ca^2+^ levels are maintained, at least in part, by extracellular Ca^2+^ influx. This is a phenomenon commonly observed in myotubes carrying pathogenic *RYR1* variants with elevated resting intracellular Ca^2+^. Under Ca^2+^-free conditions, constitutive Ca^2+^ influx is abolished, whereas cytosolic Ca^2+^ continues to be extruded through plasma membrane Ca^2+^ transport mechanisms such as the plasma membrane Ca^2+^-ATPase (PMCA) and the Na^+^/Ca^2+^ exchanger (NCX), resulting in a decline in resting intracellular Ca^2+^. In addition, caffeine-induced activation of RYR1 promotes Ca^2+^ release from the sarcoplasmic reticulum, after which a portion of the released Ca^2+^ is extruded from the cell. Although these findings suggest that abnormal Ca^2+^ influx may contribute to the altered Ca^2+^ homeostasis observed in Case 165, it remains unclear whether this mechanism is responsible for the enhanced CICR observed in this patient despite the absence of pathogenic *RYR1* variants.

Interestingly, the response to dantrolene differed from that observed in the RYR1 variant group. Dantrolene is known to suppress Ca^2+^ release primarily through inhibition of the ryanodine receptor [25]. While RYR1-variant myotubes exhibited a pronounced reduction in intracellular Ca^2+^ upon dantrolene exposure, Case 165 showed a response comparable to that of the CICR-negative group. This finding suggests that although the Ca^2+^ dysregulation phenotype in Case 165 resembles RYR1-associated MH in several aspects, the underlying molecular mechanism may not involve direct hypersensitivity of RYR1 to dantrolene. Instead, altered Ca^2+^ regulation through alternative pathways, such as sarcoplasmic reticulum stability or SOCE modulation, may be implicated.

Collectively, the findings from Figure 6, Figure 7 and Figure 8 suggest that both extracellular Ca^2+^ entry and RYR-dependent Ca^2+^ release contribute to the altered Ca^2+^ homeostasis observed in Case 165. Elevated resting intracellular Ca^2+^, its marked reduction following extracellular Ca^2+^ removal, and the lower S_1_/S_0_ ratio consistently indicate an increased dependence on extracellular Ca^2+^ entry. In contrast, the relatively limited response to dantrolene compared with the RYR1 variant group suggests that excessive RYR1 activation is unlikely to be the primary abnormality. Thus, compared with classical RYR1-associated MH, altered extracellular Ca^2+^ entry may play a relatively greater role in maintaining the abnormal Ca^2+^ homeostasis in Case 165.

Taken together, our findings support the hypothesis that genes beyond *RYR1* and *CACNA1S* may contribute to MH susceptibility [2]. The identification of *ANO5* and *ORAI3* variants in CICR-positive individuals, combined with functional evidence of Ca^2+^ dysregulation, expands the spectrum of candidate genes potentially involved in MH pathogenesis.

Several limitations should be acknowledged. First, the sample size was limited, and the variants were identified in a single family. Second, segregation analysis and in vivo validation were not performed. Third, the individual contribution of each variant could not be determined, and synergistic effects cannot be excluded. Further functional studies using gene-editing models or heterologous expression systems are required to clarify the precise pathogenic mechanisms. Because both the *ANO5* and *ORAI3* variants were identified in the affected individuals, the present study cannot determine the relative contribution of each variant to the observed calcium dysregulation and malignant hyperthermia susceptibility. The elevated resting intracellular Ca^2+^ concentration, together with its reduction following extracellular Ca^2+^ removal but not after dantrolene treatment, suggests that altered Ca^2+^ influx rather than abnormal RYR1-mediated Ca^2+^ release may contribute to the phenotype. Given the known role of ORAI3 in calcium entry pathways, the *ORAI3* variant may be involved; however, further studies are required to clarify the contribution of each variant.

## 5. Conclusions

Our findings identify *ANO5* and *ORAI3* as candidate genetic contributors in a CICR-positive individual without RYR1 mutations. These variants may be associated with altered intracellular Ca^2+^ regulation; however, further genetic and functional studies are required to determine their significance in MH susceptibility.

## Figures and Tables

**Table 1:** Clinical characteristics of the study subjects used for intracellular Ca^2+^ measurements.

	Gender	Indication for CICR Testing	Results of CICR Testing	*RYR1* Variants	*CACNA1S* Variants	*ANO5* Variants	*ORAI3* Variants
WES group							
Case 165	F	Family of Idiopathic HyperCKemia	Positive	Non	Non	p.Arg547Gln	p.Arg287Cys
RYR1 variants group							
No.1	F	MH Family CCD	Positive	p.Arg2508Cys	Non	Non	Non
No.2	M	MH Family	Positive	p.Arg2508His	Non	Non	Non
No.3	F	MH Family	Positive	p.Leu4838Val	Non	Non	Non
No.4	M	MH Family	Positive	p.Thr2206Mey	Non	Non	Non
No.5	F	MH	Positive	p.Arg2508Cys	Non	Non	Non
No.6	F	MH Family	Positive	p.Gln15Lys	Non	Non	Non
No.7	M	MH	Positive	p.Arg5025Gly	Non	Non	Non
CICR negative group							
No.1	M	MH Family	Negative	Non	Non	Non	Non
No.2	M	MH Family	Negative	Non	Non	Non	Non
No.3	M	MH Family	Negative	Non	Non	Non	Non
No.4	F	MH Family	Negative	Non	Non	Non	Non
No.5	M	Idiopathic HyperCKemia	Negative	Non	Non	Non	Non
No.6	F	MH Family	Negative	Non	Non	Non	Non
No.7	M	MH Family	Negative	Non	Non	Non	Non

**Table 2:** Summary of Ca^2+^ Imaging Data. n, number of cultured myotubes analyzed from Case 165; N, number of patients. Data for the RYR1 variant group (N = 7) and the CICR-negative group (N = 7) represent the mean values obtained from each patient.

	Case 165 (*n* = 7–13)	RYR1 Variants Group (*N* = 7)	CICR-Negative Group (*N* = 7)	Kruskal-Wallis *p* Value
EC50 Caffeine (mM)	1.68 [1.63–2.16]	2.15 [1.71–2.44]	4.64 [4.28–5.52]	<0.0001
EC50 4-CmC (µM)	118.4 [78.0–160.8]	81.1 [75.5–127.9]	243.0 [225.6–270.4]	0.0022
Resting Ca^2+^ levels (nM)	108.0 [93.6–121.7]	102.6 [100.5–119.0]	69.0 [63.1–71.8]	0.0006
Ca^2+^ reduction induced by Ca-free solution (nM)	66.4 [50.4–102.9]	68.3 [41.0–73.7]	17.9 [13.3–25.4]	0.0008
Ca^2+^ reduction induced by 50 µM dantrolene (nM)	17.0 [13.8–25.7]	29.5 [23.7–36.6]	11.8 [7.7–13.6]	0.0006

## Data Availability

The datasets used and/or analyzed during the current study are available from the corresponding author on reasonable request.
